# Cumulative effect of intrinsic capacity domains on geriatric syndromes and functionality in older inpatients

**DOI:** 10.1186/s12877-025-06697-9

**Published:** 2025-11-25

**Authors:** Nagihan Sözen Gencer, Sibel Çavdar, Fatma Ozge Kayhan Koçak, Fatma Erol, Sumru Savas

**Affiliations:** 1 Division of Geriatrics, Department of Internal Medicine, Ege University Hospital, İzmir, Türkiye; 2 Division of Geriatrics, Department of Internal Medicine, Balıkesir Atatürk City Hospital, Balıkesir, Türkiye; 3 Division of Geriatrics, Department of Internal Medicine, Izmir City Hospital, İzmir, Türkiye; 4 Division of Geriatrics, Department of Internal Medicine, Izmir Tepecik Education and Research Hospital, Health Science University, Izmir, Türkiye; 5 Division of Geriatrics, Department of Internal Medicine, Diyarbakır Gazi Yaşargil Education and Research Hospital, Diyarbakır, Türkiye

**Keywords:** Intrinsic capacity, Geriatric syndromes, Frailty, Sarcopenia, Inpatients

## Abstract

**Introduction:**

Intrinsic capacity (IC), as defined by the World Health Organization, represents the composite of an individual’s physical and mental capacities and provides a multidimensional framework for assessing older adults’ functional reserve. It includes five domains: locomotion, vitality, cognition, psychological well-being, and sensory function. This study aimed to examine the associations between IC domains and common geriatric syndromes in hospitalized older adults.

**Methods:**

In this retrospective observational study, 245 patients aged ≥ 60 years who underwent comprehensive geriatric assessment were included. IC was evaluated across five domains using standard tools: gait speed, self-reported sensory deficits, Standardized Mini-Mental State Examination (S-MMSE), Euro Quality of Life 5 Domain (EuroQOL-5D) and Mini Nutritional Assessment. The IC score ranges from 0 to 5, with higher scores indicating better intrinsic capacity, and a score ≤ 2 representing moderate–severe decline. Geriatric syndromes assessed were frailty, probable sarcopenia, falls, urinary incontinence, and functional impairments in Activities of Daily Living (ADL) and Instrumental Activities of Daily Living (IADL). Logistic regression models identified predictive IC domains, adjusting for age, sex, polypharmacy, and comorbidity.

**Results:**

Nearly 60% of participants had moderate to severe IC decline (score ≤ 2). Vitality and locomotor impairments were the most common. Cognitive and vitality impairments were strongly associated with both ADL and IADL disability (*p* < 0.001), while locomotor and vitality impairments predicted probable sarcopenia and frailty (*p* < 0.01). Cognitive impairment was linked to a nearly six-fold increased fall risk (OR = 5.86, 95% CI: 2.51–13.68, *p* < 0.001). The models showed good discrimination, with an area under the curve value of 0.723 for falls and 0.854 for ADL disability.

**Conclusions:**

IC impairments, particularly in cognition, vitality, and locomotion, are prevalent and predictive of major geriatric syndromes in hospitalized older adults. Routine IC assessment may help identify at-risk individuals and inform targeted interventions.

## Introduction

Although ageing is defined in various ways in the literature, many of these definitions focus narrowly on aspects such as biological decline or increased mortality, failing to capture its multifaceted nature. In contrast, the World Health Organization (WHO) offers a broader and more holistic perspective by introducing the concept of intrinsic capacity (IC), which encompasses the composite of an individual’s physical and mental capacities that support functional ability and healthy ageing [1]. 

Recent literature increasingly emphasizes the relevance of IC in ageing research and clinical care. A recent systematic review by Piriu et al. highlighted considerable conceptual and methodological variability on how IC is defined and measured, calling for standardized, multidimensional assessment approaches [2]. Furthermore, a meta-analysis on the WHO’s Integrated Care for Older People (ICOPE) strategy showed that IC-targeted interventions can significantly improve cognitive function and depressive symptoms in older adults [3]. In addition, a scoping review evaluating the ICOPE screening tool found substantial variation in the prevalence of IC impairments and raised concerns regarding the tool’s sensitivity and specificity, indicating the need for further validation across settings [4]. Those data support the clinical relevance of IC while also revealing critical gaps in its consistent evaluation and practical application. They underscore the need for harmonized, multidimensional tools to assess IC across diverse populations and care settings.

Ageing represents both a success, and a challenge [5]. Numerous studies have reported that declines in IC are associated with higher rates of hospitalization and increased mortality [6, 7]. Additionally, IC has been linked to a higher risk of geriatric syndromes such as falls, frailty, sarcopenia, urinary incontinence, and decreased functional capacity in various populations and clinical settings [7, 8]. These findings indicate that IC is not merely a theoretical concept, but rather a comprehensive and practical tool that can be used in geriatric practice when assessing patients. However, as highlighted by recent reviews, no universal consensus exists regarding how to calculate a total IC score, and standardized assessment tools are still lacking [9, 10]. 

According to the WHO framework, IC is assessed across five core domains: locomotion, vitality, cognitive function, psychological well-being, and sensory function [11, 12]. Numerous tools have been proposed for evaluating those domains. Cognitive capacity is commonly assessed using the Mini-Mental State Examination (MMSE), while the Mini Nutritional Assessment (MNA) is frequently used to evaluate vitality, particularly nutritional status [9, 10]. The psychological domain is often measured with the Geriatric Depression Scale, though some researchers advocate for broader well-being assessments such as the Euro Quality of Life 5 Domain (EuroQOL-5D), which includes mental health indicators [13]. Locomotion is commonly assessed using physical performance measures such as gait speed, the Timed Up and Go test, and balance tests [14]. For the sensory domain, self-reported hearing and vision complaints remain the most feasible options in large-scale or routine clinical assessments [14]. As there is diversity in measurement tools, the literature consistently highlights the need for greater standardization and validation to improve comparability and clinical utility.

Despite the growing interest in IC, there is a lack of comprehensive studies examining total IC and its domains in relation to major geriatric syndromes concurrently—particularly in hospitalized older adults [15]. So, this study aims to examine the total IC and the five domains in relation to major geriatric syndromes in hospitalized older adults. As few studies have explored this relationship in inpatient settings, particularly in middle-income countries, this study contributes to a more globally relevant understanding of IC in clinical care.

## Methods

### Participants

**Fig. 1:**
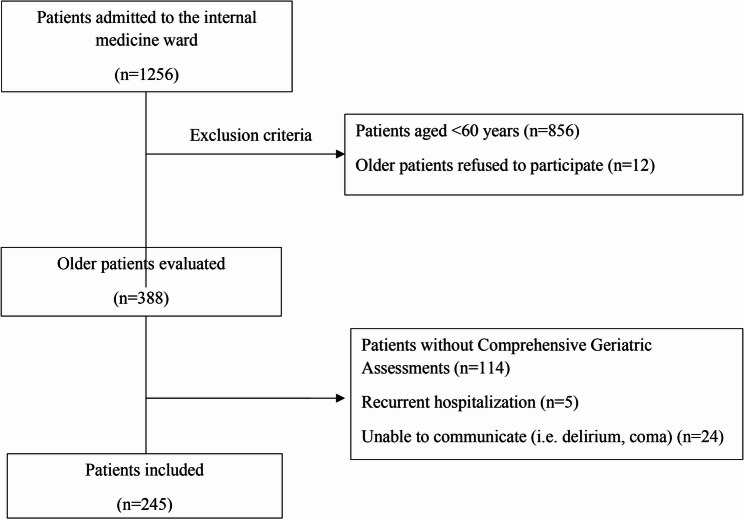
Flowchart of the study

This retrospective observational study involving older patients aged 60 years and over was conducted in the Department of Internal Medicine, Medical Faculty Hospital of Ege University, from June 2021 to September 2023. The participants’ age, sex, comorbidities, medications, socio-demographics, presence of pressure sores, Activities of Daily Living (ADLs) and the Instrumental ADLs (IADLs), domains of IC and geriatric syndromes were retrieved from the hospital records, retrospectively. Patients with data available within the first week of admission to the clinic were deemed eligible. The inclusion and exclusion criteria are explained in details in Fig. 1. A post-hoc power analysis was performed for a multiple linear regression model with eight predictors using G Power software version 3.1. With 245 samples and 8 predictors, our study has a power of 99.70%, an effect size (f2) of 0.15 with a critical 𝐹 value of 1.97 at an alpha level of 0.05.

### Measures

#### Intrinsic capacity

To assess IC, the five domains-locomotor, sensory, cognitive, psychological, and vitality-were evaluated. In our study, we chose to assess locomotion using the 4-m gait speed. Locomotion impairment (LI) was defined as gait speed *≤* 0.8 m/s [14, 16]. The sensory domain was assessed by self-reported responses for the presence of visual and hearing impairments. Participants with an answer of ‘yes’ to the question ‘Do you have any complaints about your vision or hearing’ were classified as having a sensory impairment (SI) [14]. Cognition was assessed by the Standardized Mini-Mental State Examination (S-MMSE), and cognitive impairment (CI) was defined as a S-MMSE score < 24 [17]. S-MMSE was utilized to assess cognitive function, with permission obtained from Molloy DW for use. The psychological domain was measured using the EuroQOL-5D which measures health-related quality of life in five dimensions: mobility, self-care, usual activities, pain/discomfort, and anxiety/depression. The EuroQOL-5D was used to represent psychological capacity because its anxiety/depression dimension has previously been used for this purpose in IC studies. Psychological impairment (PI) was considered to be indicated by a total score of ≥ 2 [13, 14]. The vitality domain was assessed using the MNA score. A score *≤* 11 was classified as vitality capacity impairment (VI) [18]. 

In the present study, a scoring system was developed by a multidisciplinary expert team consisting of two geriatricians and one internist who specialises in geriatric medicine. One point was assigned for each preserved domain (0–5 total). A score ≤ 2 indicates impairment in three or more domains and represents moderate–severe IC decline; a higher IC score indicates greater IC.

#### Geriatric syndromes

Frailty, falls, urinary incontinence, and probable sarcopenia as well as functional impairments were noted. Frailty was defined by the FRAIL (fatigue, resistance, ambulation, illnesses, and loss of weight) scale. FRAIL scale scores range from 0 to 5 (i.e., 1 point for each component; 0 = best to 5 = worst) and represent frail (3–5), pre-frail (1–2), and robust (0) health status [19, 20]. The assessment of fall risk was the occurrence of falls within a year. The patients were asked whether they had complaints of urinary incontinence, and evaluated as present or absent. Handgrip strength (HGS) was used for the definition of probable sarcopenia. According to EWGSOP 2, probable sarcopenia was defined as a HGS value in the dominant hand below a certain level [21]. Low HGS was determined by normative regional thresholds [22] (for males < 35 kg, and females < 20 kg) in the present study, measured by the Jamar digital dynamometer (Jamar Plus + Dynamometer, Performance Health Supply, Inc, Cedarburg, WI) validated for older adults [23]. 

#### Functionality

Functional impairment was defined by Katz Index of Independence in ADLs and the Lawton IADLs, if scores were < 5, and < 8, respectively [24–27]. 

### Statistical analysis

Normality of the data was assessed using the Kolmogorov-Smirnov test. Baseline characteristics of the study population are presented as means ± standard deviations for normally distributed continuous variables or medians and minimum-maximum values for skewed continuous data. Numbers and percentages are used for categorical variables. The chi-squared (χ2) test and Fisher’s exact test were used to compare categorical variables, while the independent sample t-test and Mann Whitney-U test were used to compare continuous variables where available. To examine which intrinsic capacity (IC) subdomains best predicted each geriatric syndrome (frailty, falls, urinary incontinence, probable sarcopenia, and disability), a series of binary logistic regression models were constructed. Five IC subdomains (locomotor, sensory, cognitive, psychological, and vitality) were initially entered as independent variables in Model 1 (full IC model). A backward stepwise selection method based on the likelihood ratio test (Backward LR) was applied to identify the most relevant predictors. The IC subdomains retained in Model 1 were subsequently included in Model 2 (selected IC model). When only two strong predictors were identified in Model 1, Model 2 was omitted and Model 3 (best combination model) was directly constructed to include the most parsimonious set of IC subdomains. All models were adjusted for age, sex, number of medications, and chronic disease count. To compare predictive performance among models, we used area under the receiver operating characteristic curve (AUC), Hosmer–Lemeshow goodness-of-fit statistics, and Nagelkerke R² values. Model discrimination was evaluated by ROC curve analysis. Correlations among non-normally distributed continuous variables were assessed using the Spearman correlation coefficient (interpreted as weak < 0.40, moderate 0.41–0.60, strong > 0.61). A two-tailed *p* < 0.05 was considered statistically significant. Analyses were performed using IBM SPSS Statistics for Windows, version 25.0.

## Results

A total of 245 patients were included (52.7% women). The mean (± SD) age of patients was 71.8 ± 8.3 years. Only one (0.4%) patient was admitted from a nursing home. Of all, 71% (*n* = 174) of the patients were hypertensive, 45.3% (*n* = 111) of the patients were with diabetes mellitus, 30.6% (*n* = 75) with cardiovascular disease, 31% (*n* = 76) with chronic kidney disease. Median total IC score was 2 (0–5). Moderate to severe decline (IC score ≤ 2) was present among almost 60% of the patients. More than half of the patients were evaluated to have LI (60.8%) or PI (51.4%). Baseline characteristics of the study participants are shown at Table 1.

**Table 1:** Baseline characteristics of study

Variable	All patients
Age, years, median (min-max)	70 (60–96)
Number of medications, median (min-max)	6 (0–19)
Number of diseases, median (min-max)	3 (0–11)
ADL score, median (min-max)	6 (0–6)
IADL score, median (min-max)	8 (0–8)
FRAIL score, median (min-max)	3 (0–5)
Handgrip strength, kg, median (min-max)	19.7 (3.8–42.4)
IC score, median (min-max)	2 (0–5)
Domains of IC	
Gait speed	
Median (min-max)	0.7 (0–1.6.6)
Locomotion impairment, n (%)	149 (60.8)
Sensory domain	
Hearing impairment, n (%)	85 (34.7)
Visual impairment, n (%)	102 (41.6)
Total sensory impairment, n (%)	196 (80)
S-MMSE	
Median (min-max)	27 (0–30)
Cognitive impairment, n (%)	42 (17.1)
EuroQOL-5D	
Median (min-max)	2 (1–3)
Psychological impairment, n (%)	126 (51.4)
MNA	
Median (min-max)	10 (0–14)
Vitality capacity impairment, n (%)	168 (68.6)
IC score group, n (%)	
0	15 (6.1)
1	51 (20.8)
2	79 (32.2)
3	67 (27.3)
4	31 (12.7)
5	2 (0.8)

*ADL* Activities of Daily Living, *IADL* Instrumental Activities of Daily Living, *FRAIL* Fatigue, Resistance, Ambulation, Illnesses, and Loss of Weight, *IC* Intrinsic Capacity, *S-MMSE* Standardized Mini-Mental State Examination, *EuroQOL-5D* Euro Quality of Life 5 Domain, *MNA* Mini Nutritional Assessment

The IC score was significantly lower in patients with pressure ulcers, ADL/IADL disability, and probable sarcopenia than in those without (*p* < 0.05). On the other hand, there was no significant difference in the IC score based on gender, education, regular income, and living alone (*p* > 0.05). The comparisons of median IC score are given at Table 2.

**Table 2:** Comparison of median IC score based on demographic variables and geriatric syndromes

Variables	*N*	%	IC score Median (min-max)	*P* value	Z score
Gender					
Female	129	52.7	2 (0–5)	0.517	−0.648
Men	116	47.3	2 (0–4)		
Education					
Illiterate	21	8.6	2 (0–4)	0.157	−1.416
Other	224	91.4	2 (0–5)		
Regular income					
Yes	218	89	2 (0–5)	0.926	−0.092
No	27	11	2 (0–4)		
Living alone					
No	204	83.3	2 (0–5)	0.598	−0.527
Yes	41	16.7	2 (0–4)		
Incontinence					
No	156	63.7	2 (0–5)	**0.006**	−2.727
Yes	89	36.3	2 (0–5)		
Falls					
No	177	72.2	2 (0–4)	**0.002**	−3.061
Yes	68	27.8	2 (0–5)		
Pressure sores					
No	243	99.2	2 (0–5)	**0.04**	−2.041
Yes	2	0.8	0 (0–1)		
ADL Disability					
No	187	76.3	2 (0–5)	**< 0.001**	−5.260
Yes	58	23.7	1 (0–4)		
IADL Disability					
No	126	51.4	3 (0–5)	**< 0.001**	−4.609
Yes	119	48.6	2 (0–5)		
FRAIL					
Non-frail	29	11.8	3 (1–4)	**< 0.001**, Frail < Prefrail	*41.842
Prefrail	74	30.2	3 (1–4)	**< 0.001**, Frail < Non-frail	*79.926
Frail	143	58	2 (0–5)	**0.03**, Prefrail < Non-frail	*38.084
Probable Sarcopenia					
No	53	21.6	3 (0–4)	**0.001**	−3.179
Yes	192	78.4	2 (0–5)		

*Kruskal-Wallis H

The Mann–Whitney U test was used for two-group comparisons and the Kruskal–Wallis test for comparisons involving more than two groups

*ADL* Activities of Daily Living, *IADL* Instrumental Activities of Daily Living, *IC* Intrinsic Capacity

After univariate analyses, the variable representing the number of patients with pressure ulcers was excluded from multivariate logistic regression due to insufficient sample size (< 10 cases). In the multivariate model 1 showed that CI was significantly associated with ADL disability (OR 9.67, *p* < 0.001), IADL disability (OR 10.75, *p* < 0.001), and falls (OR 5.39, *p* < 0.001), whereas LI was significantly associated with probable sarcopenia (OR 2.70, *p* = 0.008) and frailty (OR 3.51, *p* = 0.01). While SI was only associated with disabilities and falls, PI was not associated with any of the geriatric syndromes. In the final Model 3, the locomotor and vitality subdomains were significantly associated with probable sarcopenia (locomotor; OR = 2.59, 95% CI: 1.28–5.24, *p* = 0.008 and vitality; OR = 2.72 95%, CI: 1.34–5.23, *p* = 0.006) and frailty (locomotor; OR = 3.94, 95% CI: 1.57–9.85, *p* = 0.003 and vitality; OR = 5.41, 95% CI: 2.16–13.53, *p* < 0.001). The cognitive and vitality subdomains showed strong associations with ADL disability (cognitive; OR = 8.50, 95% CI: 3.41–21.16, *p* < 0.001and vitality; OR = 8.23, 95% CI: 2.46–27.50, *p* = 0.001) and IADL disability (cognitive; OR = 10.50, 95% CI: 2.92–37.72, *p* < 0.001 and vitality; OR = 2.77, 95% CI: 1.44–5.31, *p* = 0.002). The sensory and cognitive domains were significantly associated with falls, and the cognitive domain was associated with increased risk of falls (OR = 5.86, 95% CI: 2.51–13.68, *p* < 0.001). Logistic regression results are presented in Table 3.

**Table 3:** Logistic regression results for geriatric syndromes

	Probable sarcopenia		ADL disability		
Predictor	Model 1 OR (95%Cl), *p*	Model 3 OR (95%Cl), *p*	Model 1 OR (95%Cl), *p*	Model 2 OR (95%Cl), *p*	Model 3 OR (95%Cl), *p*
**LI**	2.70 (1.29–5.63), **0.008**	2.59 (1.28–5.24), **0.008**	1.73 (0.72–4.15), 0.22	-	-
**SI**	0.39 (0.14–1.08), 0.07	-	0.34 (0.13–0.87), **0.02**	0.39 (0.16–0.96), **0.04**	-
**CI**	2.98 (0.77–11.50), 0.11	-	9.67 (3.72–25.15), **< 0.001**	9.65 (3.76–24.74), **< 0.001**	8.50(3.41–21.16), **< 0.001**
**PI**	1.01 (0.50–1.05), 0.98	-	1.16 (0.53–2.53), 0.72	-	-
**VI**	2.76 (1.33–5.72), **0.007**	2.72 (1.34–5.23), **0.006**	9.34 (2.73–31.98), **< 0.001**	8.75 (2.61–29.32), **< 0.001**	8.23 (2.46–27.50), **0.001**
	Frailty *		**IADL disability**		
	**Model 1** **OR (95%Cl)**,** p**	**Model 3** **OR (95%Cl)**,** p**	**Model 1** **OR (95%Cl)**,** p**	**Model 2** **OR (95%Cl)**,** p**	**Model 3** **OR (95%Cl)**,** p**
**LI**	3.51 (1.33–9.25), **0.01**	3.94 (1.57–9.85), **0.003**	1.31 (0.69–2.48), 0.4	-	-
**SI**	0.36 (0.07–1.75), 0.2	-	0.39 (0.18–0.85), **0.02**	0.40 (0.18–0.86), **0.02**	-
**CI**	-	-	10.75 (2.96–39.05), **< 0.001**	11.52 (3.17–41.86), **< 0.001**	10.50(2.92–37.72), **< 0.001**
**PI**	2.75 (1.01–7.50), 0.05	-	1.23 (0.67–2.27), 0.5	-	-
**VI**	4.54 (1.80–11.41.80.41), **0.001**	5.41(2.16–13.53), **< 0.001**	3.03 (1.54–5.96), **0.001**	3.01 (1.54–5.89), **0.001**	2.77 (1.44–5.31), **0.002**
	**Falls**		**Urinary incontinence**		
	**Model 1** **OR (95%Cl)**,** p**	**Model 3** **OR (95%Cl)**,** p**	**Model 1** **OR (95%Cl)**,** p**		
**LI**	1.42 (0.72–2.80), 0.3	-	1.02 (0.55–1.88), 0.9		
**SI**	0.44 (0.21–0.92), **0.03**	0.46 (0.22–0.96), **0.04**	1.17 (0.56–2.42), 0.7		
**CI**	5.39(2.29–12.66), **< 0.001**	5.86(2.51–3.68), **< 0.001**	1.58(0.71–3.50), 0.3		
**PI**	1.45(0.76–2.73), 0.26	-	1.50(0.84–2.68), 0.2		
**VI**	1.16(0.58–2.34), 0.7	-	1.15(0.61–2.17), 0.17		

Model 1 was included all IC domains. Model 2 was included the selected domains from Model 1. Model 3 was included the best combination of domains

All models were adjusted for age, sex, the number of medications, and diseases. P-value significant (i.e., *≤* 0.05) indicated in bold

*LI* Locomotion Impairment, *SI* Sensory Impairment, *CI* Cognitive Impairment, *PI* Psychological Impairment, *VI* Vitality Impairment, *OR* Odds Ratio, *CI* Confidence Interval

* Frailty versus prefrail + robust

The overall model performance for each geriatric syndrome in Model 3, shown in Table 4, showed good discriminatory ability, with AUC values ranging from 0.723 (falls) to 0.854 (ADL disability), and acceptable calibration based on the Hosmer–Lemeshow goodness-of-fit test (all *p* > 0.05). Model fit, as assessed by the Nagelkerke R² statistic, indicated that the IC subdomains explained a substantial proportion of the variance in ADL disability (R²=0.462), IADL disability (R²=0.360), and frailty (R²=0.304). Probable sarcopenia and falls had lower explanatory values (R²=0.272 and 0.212, respectively), suggesting that while IC subdomains contribute meaningfully to these outcomes, additional factors may also play a role.

**Table 4:** Comparative logistic regression model 3 for geriatric syndromes

Geriatric syndrome	IC Subdomains included	AUC (95%Cl)	Nagelkerke *R*^2^	Hosmer and Lemeshow test *p*
**Probable sarcopenia**	Locomotor and vitality	0.789 (0.727–0.852)	0.272	0.39
**ADL disability**	Cognitive and vitality	0.854 (0.794–0.913)	0.462	0.507
Frailty*	Locomotor and vitality	0.813 (0.730–0.896)	0.304	0.169
**IADL disability**	Cognitive and vitality	0.777	0.360	0.410
**Falls**	Sensory and cognitive	0.723	0.212	0.930

* Frailty versus prefrail + robust

In Spearman correlation analysis, the IC score is correlated with age, ADL score, IADL score, FRAIL score, and HGS (*p* < 0.001). Except for the IC domains, FRAIL score was moderately correlated with IC scores (*r*=−0.462, *p* < 0.001 respectively). Addressing at the domains separately, IADL scores were strongly correlated with the cognitive domain (*r* = 0.624, *p* < 0.001). The correlation analysis is shown in Table 5.

**Table 5:** Spearman correlation between IC and comprehensive geriatric assessment

		IC score	Age	ADL	IADL	FRAIL	HGS	Gait speed	S-MMSE	EuroQOL	MNA-SF
**IC score**	**r**	1	-,231**	,376**	,356**	-,462**	,338**	,553**	,346**	-,553**	,528**
**Age**	**r**	-	1	-,405**	-,418**	,230**	-,317**	-,336**	-,359**	,008	-,159*
**ADL**	**r**	-	-	1	,705**	-,506**	,482**	,330**	,441**	-,199*	,408**
**IADL**	**r**	-	-	-	1	-,533**	,510**	,303**	,624**	-,131*	,420**
**FRAIL**	**r**	-	-	-	-	1	-,507**	-,384**	-,424**	,203**	-,539**
**HGS**	**r**	-	-	-	-	-	1	,293**	,507**	-,125	,373**
**Gait speed**	**r**	-	-	-	-	-	-	1	,266*	-,180*	,185*
**S-MMSE**	**r**	-	-	-	-	-	-	-	1	-,077	,319**
**EuroQOL**	**r**	-	-	-	-	-	-	-		1	-,202**
**MNA-SF**	**r**	-	-	-	-	-	-	-	-	-	1

*ADL* Activities of Daily Living, *IADL* Instrumental Activities of Daily Living, *FRAIL* Fatigue, Resistance, Ambulation, Illnesses, and Loss of Weight, *HGS* Handgrip Strength, *IC* Intrinsic Capacity, *S-MMSE* Standardized Mini-Mental State Examination, *EuroQOL-5D* Euro Quality of Life 5 Domain, *MNA-SF* Mini Nutritional Assessment Short Form

* Correlation is significant at the 0.05 level (2-tailed)

** Correlation is significant at the 0.001 level (2-tailed)

## Discussion

In our study on hospitalized older adults, we explored the relationships between IC and common geriatric syndromes by evaluating the predictive values of five IC subdomains. Our findings revealed that impairments in IC were highly prevalent, with nearly 60% of patients exhibiting moderate to severe IC decline (IC score ≤ 2). LI and VI were the most common subdomain deficits, and these domains, along with cognition, showed significant associations with frailty, probable sarcopenia, and functional impairments in both basic and instrumental ADLs. Notably, CI emerged as a strong predictor of ADL/IADL disability and falls, while VI was consistently associated with all functional outcomes assessed. These results reinforce the utility of IC as a comprehensive, clinically relevant construct in geriatric assessment and align with previous evidence suggesting that targeted assessment of IC domains may offer valuable insights into an older adult’s functional trajectory and care needs.

Individuals with low IC at baseline were shown to develop frailty approximately 1.57 times more compared to those with preserved IC [28]. In our study, the associations between IC subdomains and geriatric syndromes largely coincided with previous studies in different populations, but also presented some specific findings. Locomotor and vitality were found to be the IC domains most strongly associated with frailty. This result is consistent with a study on 4000 community-dwelling individuals conducted in 2022, which reported that individuals with higher physical performance and better nutritional status were significantly less likely to develop frailty [29]. Furthermore, recently Yu et al. emphasized that vitality capacity is the physiological basis of other IC subdomains and that a decrease in energy reserve may be an early indicator of pre-frailty [30]. In 2023, Shen et al. found a significant relationship between frailty and cognitive, psychological, locomotor, vitality, and vision loss in hospitalized patients, whereas our study found no statistically significant relationship between psychological and sensory domains and frailty [31]. This difference may be due to sample size, patient comorbidities, reasons for hospitalization, measurement tools for IC subdomains, or differences in the timing of assessment. These areas may affect the development of frailty over time through indirect mechanisms such as social isolation and physical inactivity which might not be observed in hospitalized patients [32, 33]. 

The likelihood of developing disability is approximately 1.8 times higher for individuals with low IC at baseline than for those with preserved IC [28]. Moreover, each unit decline in IC has been associated with an 8% increased risk of ADL disability [34]. People with high IC were found to have significantly lower odds of functional decline—by 44% for ADLs and 51% for IADLs—compared to individuals with low IC [35]. Although no significant association was observed between IC trajectories and changes in ADL, maintaining or improving IC over time was associated with a lower risk of IADL decline [35]. These findings are consistent with our results, which demonstrated a strong association between IC subdomains and functional independence. Meta-analyses have also indicated that impairments in specific IC domains are linked to greater IADL disability [34]. In particular, declines in cognitive function are significantly associated with increased risk of both ADL and IADL disabilities [34]. In our study, impairments in the cognitive, and vitality domains were significantly associated with increased dependency in both ADLs and IADLs. Cognitive deficits—especially in executive function, attention, and memory—can impair an individual’s ability to plan and perform daily tasks. These two IC domains are therefore critical for preserving functional independence and should be routinely evaluated, especially during hospital admissions [36, 37]. Early assessment and targeted interventions in the cognitive and vitality domains may help maintain independence and improve the quality of life of older adults during and after hospitalization.

In our study, it was observed that the IC score was lower in individuals with a history of falls. Likewise, in the ilSIRENTE study, it was shown that the risk of falls was significantly lower in individuals with higher total IC levels, and it was emphasized that locomotor capacity was the component most closely related to falls [38]. In another recent study conducted by Shen et al. in hospitalized patients, falls were observed more frequently in those with low IC and the impairments in cognitive, vitality, locomotion, and psychological domains were found to be associated with the risk of falls [31]. In our study, among the components of IC, cognitive capacity showed the strongest relationship with falls. It was observed that the risk of falls increased approximately six times in individuals with low cognitive capacity. Declines in cognitive capacity increase the likelihood of falls by impairing the ability to detect environmental hazards and respond appropriately, due to deficits in attention, judgment, and executive functioning [39]. In contrast, vitality, locomotor, and psychological domains were not found to be significantly associated with falls in our study. This difference may be due to the multifactorial nature of falls, different IC assessment methods, and the number of patients [40, 41]. In our study, lower sensory capacity was unexpectedly associated with a lower likelihood of falls, which contrasts with the well-established role of sensory impairment as a risk factor for falling [42]. This counterintuitive finding may reflect methodological and behavioral factors rather than a true protective effect. As sensory impairment was self-reported, individuals who were aware of their deficits might have adopted more cautious behaviors or implemented compensatory strategies, such as using assistive devices or modifying their environment, thereby reducing their fall risk. Moreover, we did not assess key variables such as fear of falling or the use of walking aids, which could partly account for this observation. Given the retrospective design and potential residual confounding, these results should be interpreted with caution rather than implying a causal or protective relationship.

In our study, psychological capacity was not significantly associated with any of the geriatric syndromes assessed. Avcı et al. found a relationship between depressive symptoms and sarcopenia and malnutrition in their study [43]. The effects of psychological state on physical inactivity, social isolation, and loss of appetite typically emerge over the long term and may explain this inconsistency. These delayed or indirect effects may be better captured in longitudinal designs. Additionally, stress experienced during hospitalization, the current disease burden, and environmental changes may temporarily affect an individual’s mood, causing their psychological capacity to appear more variable than it actually is [44, 45]. In a recent systematic review published by Sale et al., a decline in IC was found to be significantly associated with sarcopenia [8]. Consistent with the existing literature, our study found that the IC score was significantly lower among individuals with probable sarcopenia. Similarly, Zhu et al. (2023) reported that locomotion, cognition, and vitality were positively associated with HGS [46]. In our study, the IC subdomains most associated with sarcopenia, such as frailty, were vitality and locomotor capacity [21]. The low level of vitality suggests that processes such as malnutrition, decreased energy reserves, and systemic inflammation may play a role in the development of sarcopenia [47, 48]. The lack of a significant relationship between cognitive, sensory and psychological capacity and probable sarcopenia, and the fact that sarcopenia is mainly related to muscle strength, muscle mass, and physical performance suggest that these components might be effective in the development of sarcopenia through indirect mechanisms over time [8]. The concentration of IC subdomains associated with sarcopenia, especially in the and vitality domains, also suggests that multidisciplinary approaches to support muscle strength and prevent nutritional deficiencies should be prioritized in the interventions for sarcopenia.

### Limitations

This study has some limitations. First, the study was conducted in a single hospital setting, which may limit the generalizability of the findings to broader populations. The retrospective design also prevents the establishment of causal relationships between intrinsic capacity (IC) and geriatric syndromes. Secondly, the study was conducted in a hospital setting that might have affected the influence of environmental stressors on the assessment of some IC subdomains, especially psychological and sensory capacity which might have led to underestimation of some relationships. Third, some measures for IC subdomains and geriatric syndromes such as falls, urinary incontinence are based on self-report and are at risk of response bias. In addition, patients with non-advanced dementia and Parkinson’s disease were included in the study. This may have led to some confounding effects, especially in the assessment of cognitive and locomotor domains. Moreover, the small number of participants in certain subgroups (e.g., patients with pressure ulcers) limited the statistical power of the multivariable analyses. To analyze the relationship between these neurological diseases and IC more accurately and in detail, it would be more appropriate to address these individuals in separate studies.

## Conclusion

This study demonstrates that impairments in IC are highly prevalent among hospitalized older adults and are significantly associated with key geriatric syndromes, including frailty, probable sarcopenia, falls, urinary incontinence, and functional decline. Among the five IC subdomains, locomotion, vitality, and cognition were the strongest predictors of these geriatric syndromes. Our findings highlight the value of IC as a practical and multidimensional framework for identifying older inpatients at risk for adverse health outcomes. Incorporating routine IC assessment into clinical practice may support earlier detection of vulnerability, enable targeted interventions, and contribute to the development of more individualized and preventive care strategies for older adults in acute care settings.

### Ethics approval

## Data Availability

There are ethical restrictions on publicly sharing a de-identified dataset due to sensitive patient information. Data is available from the secretary of the Ege University Ethics Committee via email (egetaek@gmail.com) for researchers who meet the criteria for access to confidential data. The datasets used and/or analysed during the current study available from the corresponding author on reasonable request with permission of Ege University Ethics Committee.
